# Editorial: Novel insights about subchondral bone remodeling in arthropathies

**DOI:** 10.3389/fphys.2024.1369928

**Published:** 2024-02-01

**Authors:** Chuan Yang, Zihan Deng, Gang Huang, Yueqi Chen

**Affiliations:** ^1^ Department of Orthopedics, Southwest Hospital, Third Military Medical University (Army Medical University), Chongqing, China; ^2^ Department of Biomedical Materials Science, Third Military Medical University (Army Medical University), Chongqing, China; ^3^ Department of Biochemistry and Molecular Biology, College of Basic Medical Science, Third Military Medical University (Army Medical University), Chongqing, China

**Keywords:** subchondral bone, arthropathies, bone homeostasis, osteoarthritis, rheumathoid arthritis, spondyloarthritis

Arthropathies, a series of diseases that are involved in inflammatory, degenerative, metabolic, and traumatic conditions, commonly manifest symptoms such as joint pain, stiffness, swelling, and movement restriction, which often result in disability and reduce the life quality of patients. Many common diseases including rheumatoid arthritis (RA), osteoarthritis (OA), temporomandibular joint osteoarthritis (TMJOA), and spondyloarthritis (SpA) come under arthropathies, which have placed an enormous burden on society. Previous studies attributed the pathogenesis of arthropathies to cartilage and synovium alterations, however, increasing research demonstrates that subchondral bone remodeling may perform an important role in these disorders.

Subchondral bone is a layer of bone that exists below the joint cartilage and provides essentially structural and mechanical support to the overlying cartilage. Notably, as a complex and dynamic architectural unit, subchondral bone consists of a specialized matrix that houses osteoblasts (OBs), osteoclasts (OCs), osteocytes, and immune cells. Immune cells and a variety of cytokines within this microenvironment allow for repairing minor damage in cartilage and facilitate communication between subchondral bone and cartilage. In addition, the presence of a network of blood vessels in subchondral bone supplies enough nutrients and oxygen for cartilage, which maintains the self-repairing capability of cartilage. The remodeling of subchondral bone, which is related to OCs and OBs that participate in the modulation of deposition and resorption in bone, may malfunction during arthropathies, thereby contributing to an abnormally biochemical and morphological transition in the subchondral bone. These alterations including subchondral bone sclerosis and osteophyte formation directly affect the homeostasis of the overlying cartilage, leading to joint pain and cartilaginous degeneration. Furthermore, vascular invasion in the subchondral bone can aggravate the arthritic condition by perpetuating a cycle of inflammation and degeneration. Therefore, elucidating the intricacies of the crosstalk between subchondral bone remodeling and joint cartilage is indispensable, which may provide a more holistic treatment approach for patients suffering from arthropathies.

This Research Topic is composed of bibliometric analysis, magnetic resonance imaging (MRI) evaluation model of SpA, potential new drug development of arthropathies, small molecule inhibitor development, anti-nerve growth factor (NGF) antibody application, as well as animal model construction research in OA, which covers a comprehensive range of different aspects related to arthropathies research including integrating cutting-edge technologies, innovative therapeutic strategies, biomedical engineering, and fundamental biological science. As editors, we found immense satisfaction in evaluating the engaging research papers and comprehensive reviews. This editorial intends to encapsulate the key discoveries and perspectives gleaned from the contributions that met our acceptance criteria.

Wen et al. performed a comprehensive bibliometric analysis on subchondral bone research, which analyzed and identified the key research trends and hotspots in OA, including inflammation, cartilage degeneration/repair, stromal cells, and mesenchymal stem cells. Their study highlighted the importance of investigating related pathological mechanisms and potential therapeutic targets of subchondral bone in OA.

Hip involvement is a common complication in SpA patients and always manifests as intractable pain induced by subchondral bone marrow edema (BME). Conventional MRI assessments of hip inflammation require enough clinical experience that cannot achieve a complete appraisal of therapeutic outcomes. Zheng et al. applied a deep learning approach to evaluate MRI scans for inflammatory changes signifying hip involvement, thereby constructing a fully automatic and visualized diagnostic system based on U-Net, which may contribute to the formulation of personalized treatment strategies for SpA patients.

As a common early symptom of ankylosing spondylitis, sacroiliitis is characterized by an inflammation of one or both of the sacroiliac joints situated where the lower spine and pelvis connect. Prati et al. discussed the anatomical, histological, and immunohistology observations related to the development of sacroiliitis, with a focus on imaging-based information. They observed subchondral abnormalities in the bone marrow of the sacroiliac joint (SIJ) including chondroid subchondral metaplasia, bone and cartilage degradation, fibrous and fatty marrow, and the expansion of chondroid structures into the articular cartilage, which promoted the understanding of subchondral bone involvement in SpA.

OA animal models play an important role in exploring the related pathophysiology and the efficacy and safety of novel therapies for OA. Joint instability surgery could mimic clinical features of OA such as subchondral bone and cartilage changes. Menges et al. demonstrated that increased movement of animals in colony cages results in robust structural alterations in subchondral bone, which contributed to the design of chronic OA studies within a longer lifespan.

Subchondral bone remodeling during OA progression stimulates the growth of new nerves into the remodeled areas, which directly links structural changes to the sensation of pain. As a growth factor for nerve cells, NGF performs important functions in OA-related acute and chronic pain. Menges et al. demonstrated that anti-NGF treatment could effectively improve OA-induced pain symptoms while worsen structures of subchondral bone and cartilage. Their study enriched comprehension of the system anti-NGF approach and facilitated the development of innovative therapeutic modalities for OA.

Current treatment options for OA are limited, mainly including NSAIDs and Glucocorticoids, with frequent and serious adverse reactions. Liu et al. focused on small molecule inhibitors that target cartilage degeneration in OA and discussed the potential of optimizing the dosage form of traditional drugs, which may improve the treatment options for OA patients.

The water and ethanol extracts of *Terminalia chebula* have been proved to possess significant antiarthritic effects. Liu et al. explored the anti-inflammatory and antioxidant effects of polyphenol extracts from *Terminalia chebula*, and identified the key role of chebulanin and chebulagic acid in these anti-arthritic components. Their study paved the way for novel treatments for arthritic conditions based on traditional asian medicine.

**FIGURE 1:**
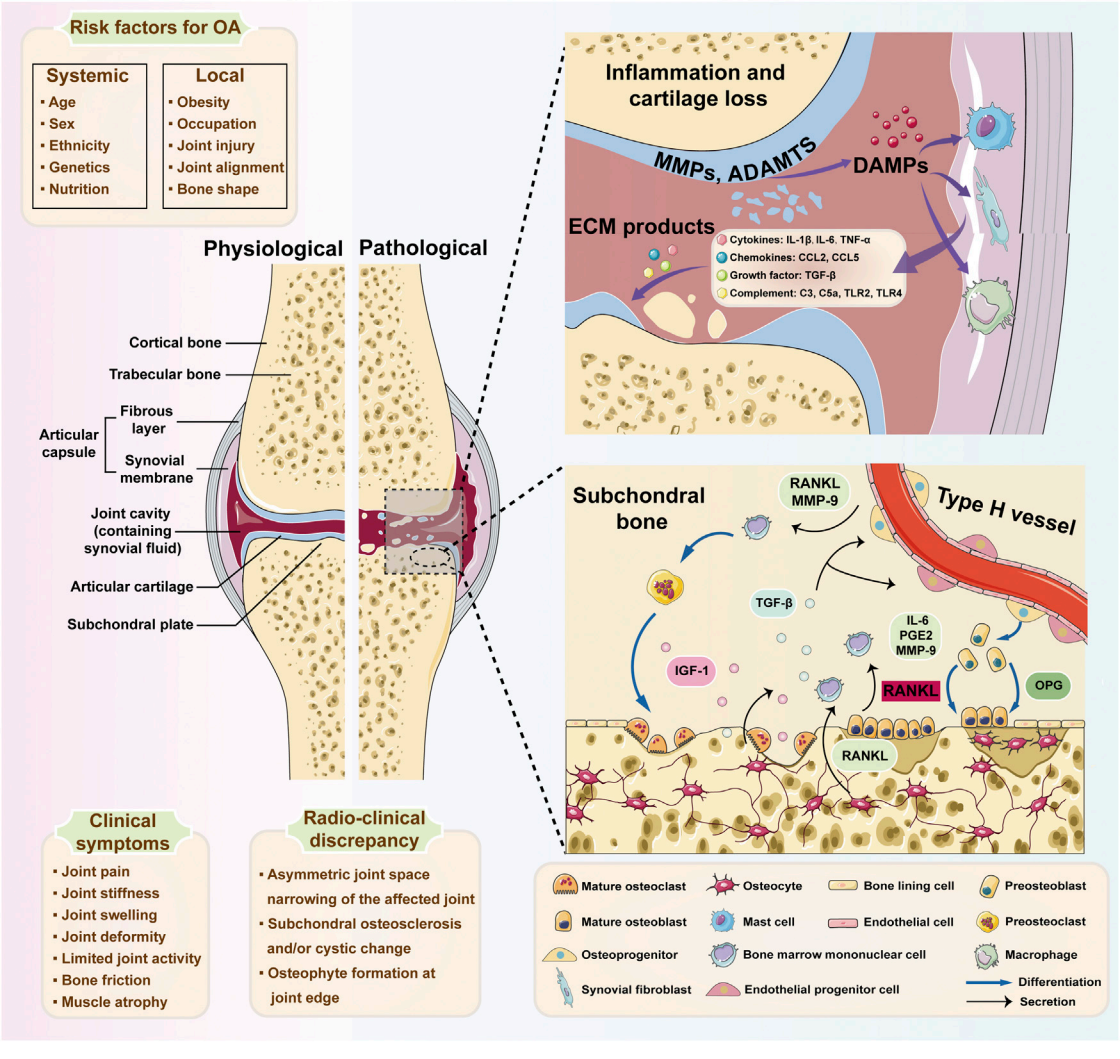
The inflammatory network and pathological cellular interactions in osteoarthritis (OA). Specific systemic risk factors (e.g., age, sex, and race) and local factors (e.g., obesity and joint injury) are considered to contribute to damage in OA. The breakdown byproducts of cartilage and extracellular matrix (ECM) constituents are discharged into the joint space as damage-associated molecular patterns (DAMPs), subsequently triggering synovial cells and macrophages to produce diverse inflammatory mediators, including cytokines, chemokines, and complement. These inflammatory molecules additionally contribute to the deterioration of chondrocyte function and the damage of cartilage. With the progression of OA, osteocytes upregulate the ratio of RANKL/OPG to facilitate osteoclast differentiation. Meanwhile, IL-6, PGE2, and MMP-9 from the osteoblasts mediate the pro-osteoclastic effect. Additionally, osteoclast-mediated bone resorption is mainly responsible for angiogenesis and osteogenesis by released TGF-β1. And RANKL and MMP-9 generated by type H endothelial cells may promote osteoclast chemotaxis and formation. MMPs, matrix metalloproteinases; ADAMTSs, a disintegrin and metalloproteinase with thrombospondin motifs; ECM, extracellular matrix; DAMPs, damage-associated molecular patterns; IL-1β, interleukin-1beta; IL-6, interleukin-6; TNF-α, tumor necrosis factor-alpha; CCL2, chemokine (C-C motif) ligand 2; CCL5, chemokine (C-C motif) ligand 5; TGF-β, transforming growth factor-beta; TLR2, Toll-like receptor 2; TLR4, Toll-like receptor 4; IGF-1, insulin-like growth factor; RANKL, receptor activator of nuclear factor κB ligand; OPG, osteoprotegerin; PGE2, prostaglandin E2. Schematic Figure 1 is created with the help of Servier Medical Art, provided by Servier, licensed under a Creative Commons Attribution 3.0 Unported License (https://smart.servier.com).

In conclusion, these studies included in this Research Topic demonstrated comprehensive understanding of subchondral bone remodeling in arthropathies progression and emphasized the significance of interdisciplinary approaches such as cutting-edge molecular biology, imaging techniques, and therapeutic strategies in the arthropathies research field. It is believed that future research directions may be involved in further investigation into the molecular mechanisms of cell interactions, the identification of specific biomarkers for early subchondral bone remodeling, and the development of targeted therapies. Collectively, this Research Topic provides valuable insights into the essential role of subchondral bone in arthropathies progression (Figure 1).

